# Supplementary figures and images for: miR-200a-3p overexpression alleviates diabetic cardiomyopathy injury in mice by regulating autophagy through the FOXO3/Mst1/Sirt3/AMPK axis
Source: PeerJ. 2023 Sep 15;11:e15840. doi: 10.7717/peerj.15840 (PMC10506579; doi:10.7717/peerj.15840)

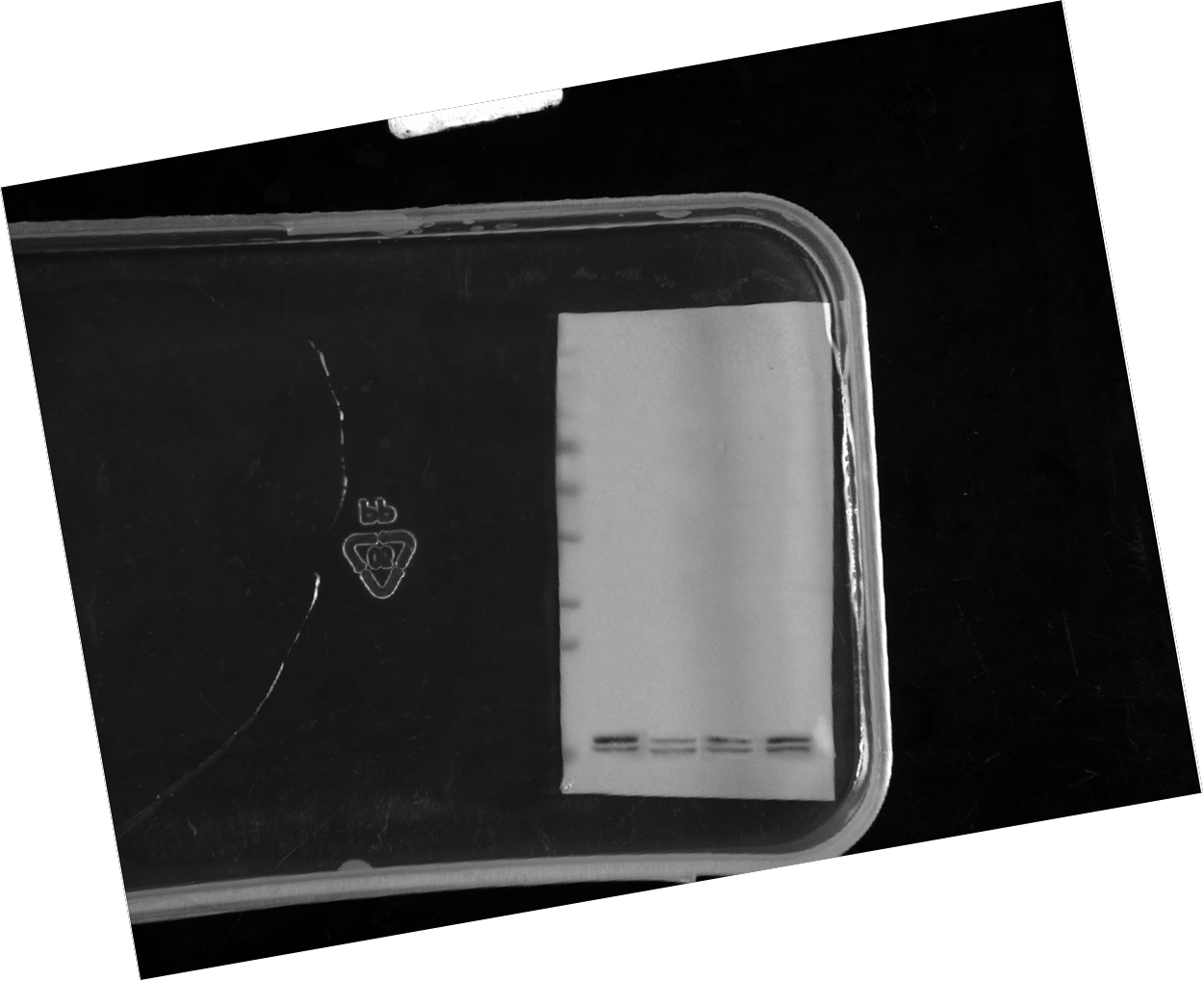

Supplement: Supplemental Information 2 [file peerj-11-15840-s002.zip › Western blot/figure 4 ( light chain 3) LC3/Original.tif]

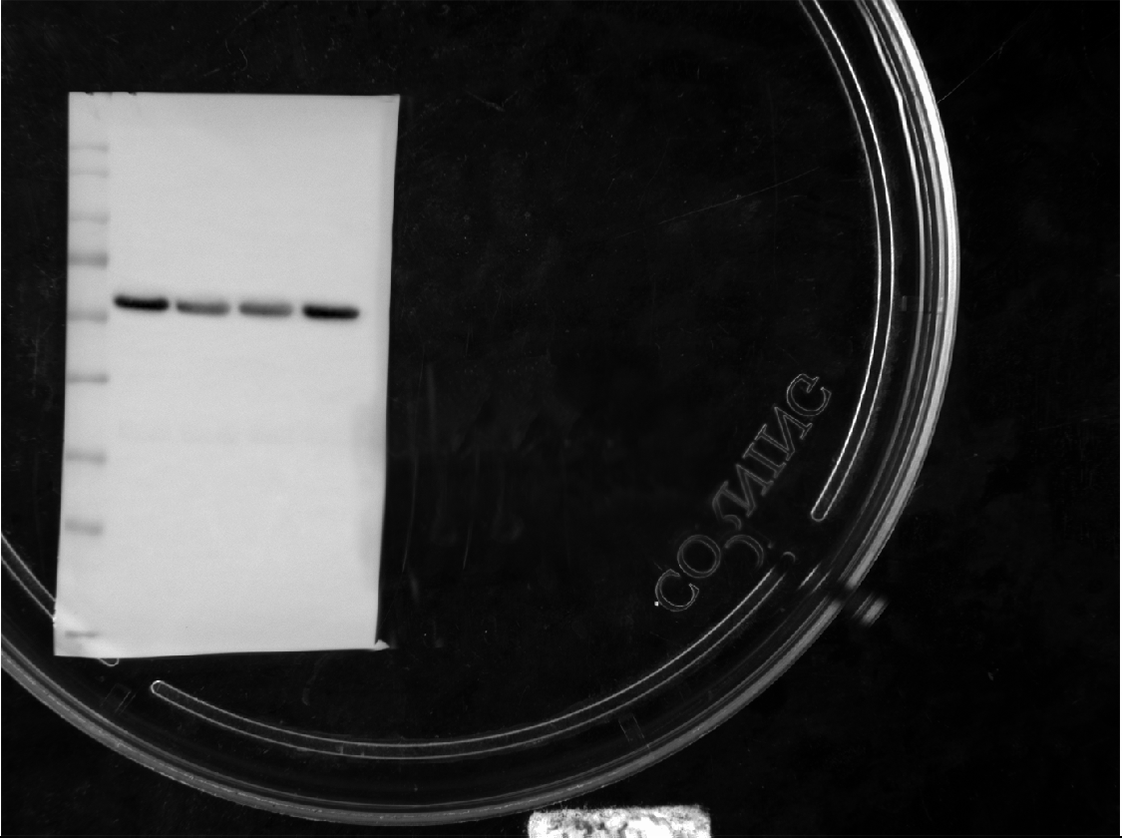

Supplement: Supplemental Information 2 [file peerj-11-15840-s002.zip › Western blot/figure 4 Beclin1/Original.tif]

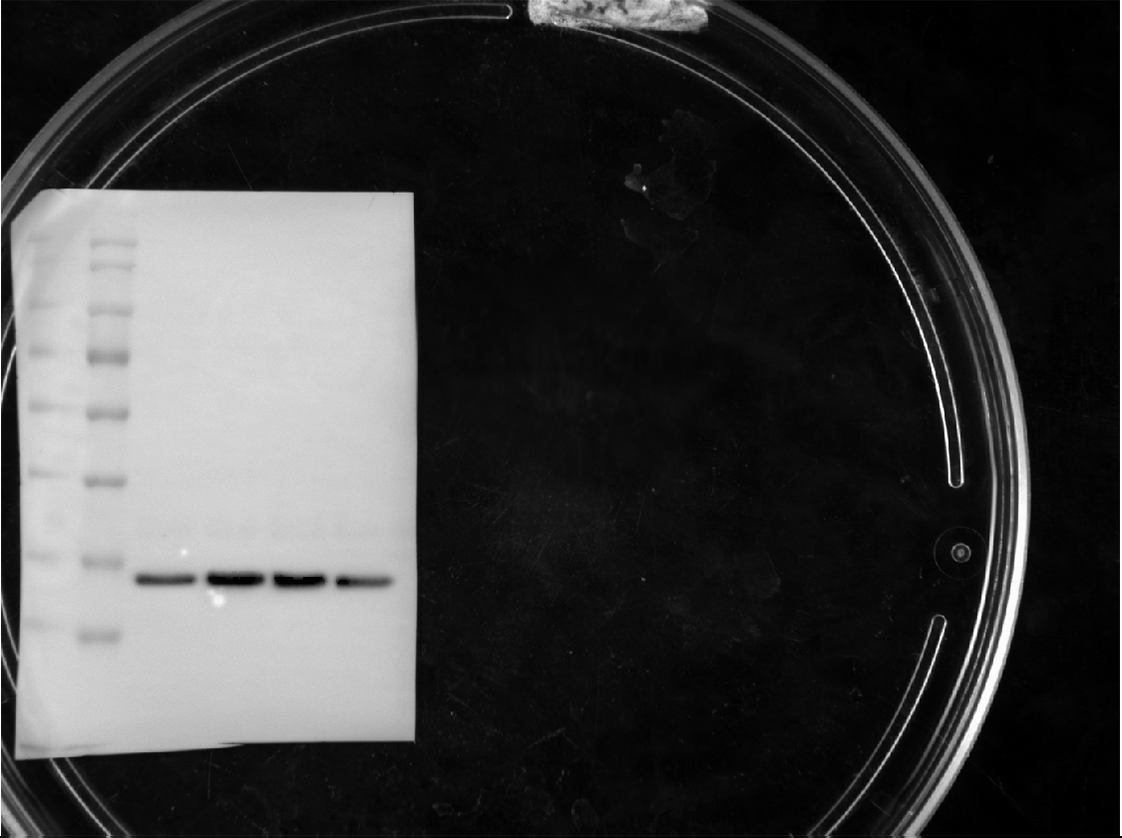

Supplement: Supplemental Information 2 [file peerj-11-15840-s002.zip › Western blot/figure 4 caspase3/Original.tif]

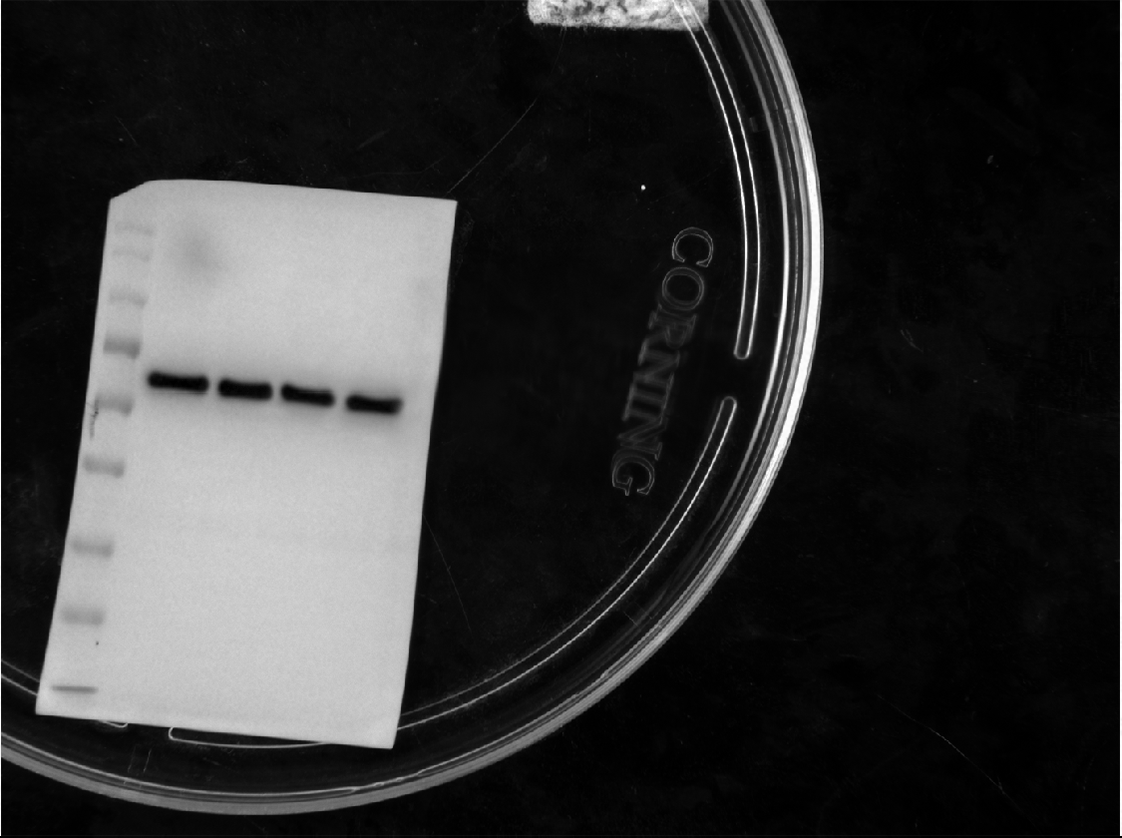

Supplement: Supplemental Information 2 [file peerj-11-15840-s002.zip › Western blot/figure 5 Adenosine 5-monophosphate (AMP)-activated protein kinase (AMPK)/Original.tif]

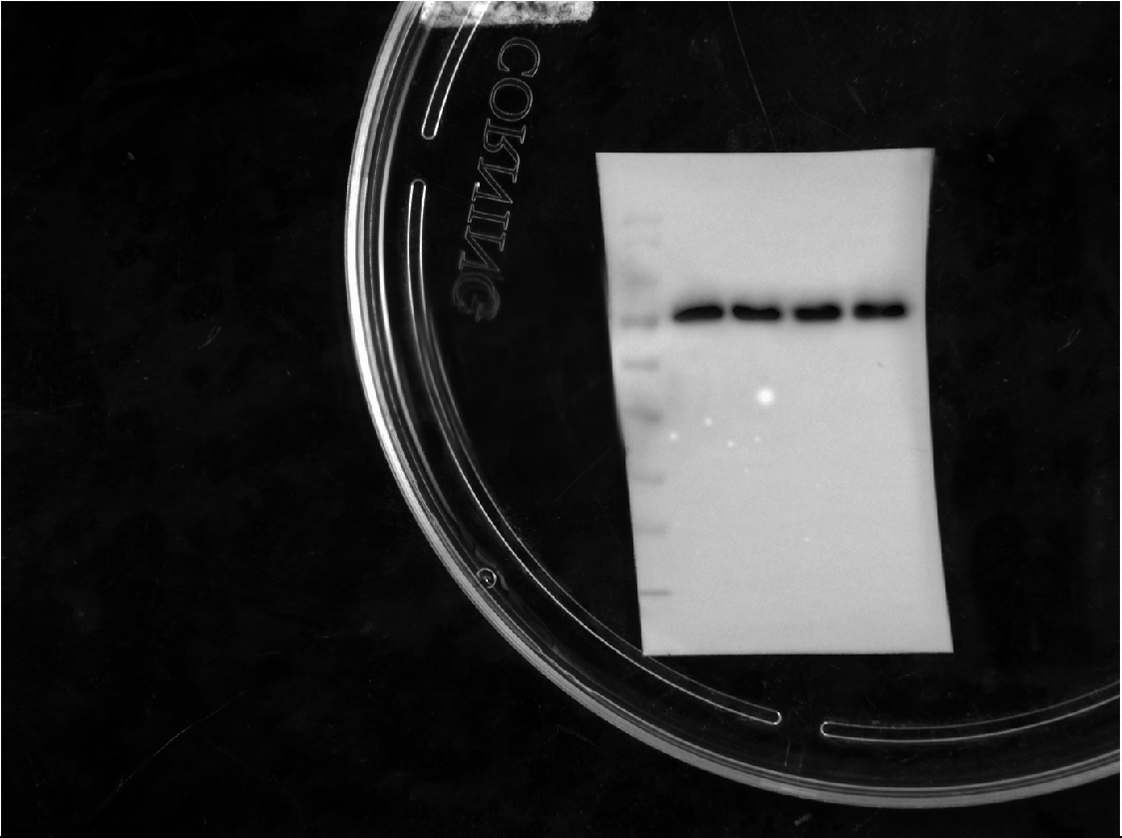

Supplement: Supplemental Information 2 [file peerj-11-15840-s002.zip › Western blot/figure 5 Forkhead box O3 (FOXO3)/Original.tif]

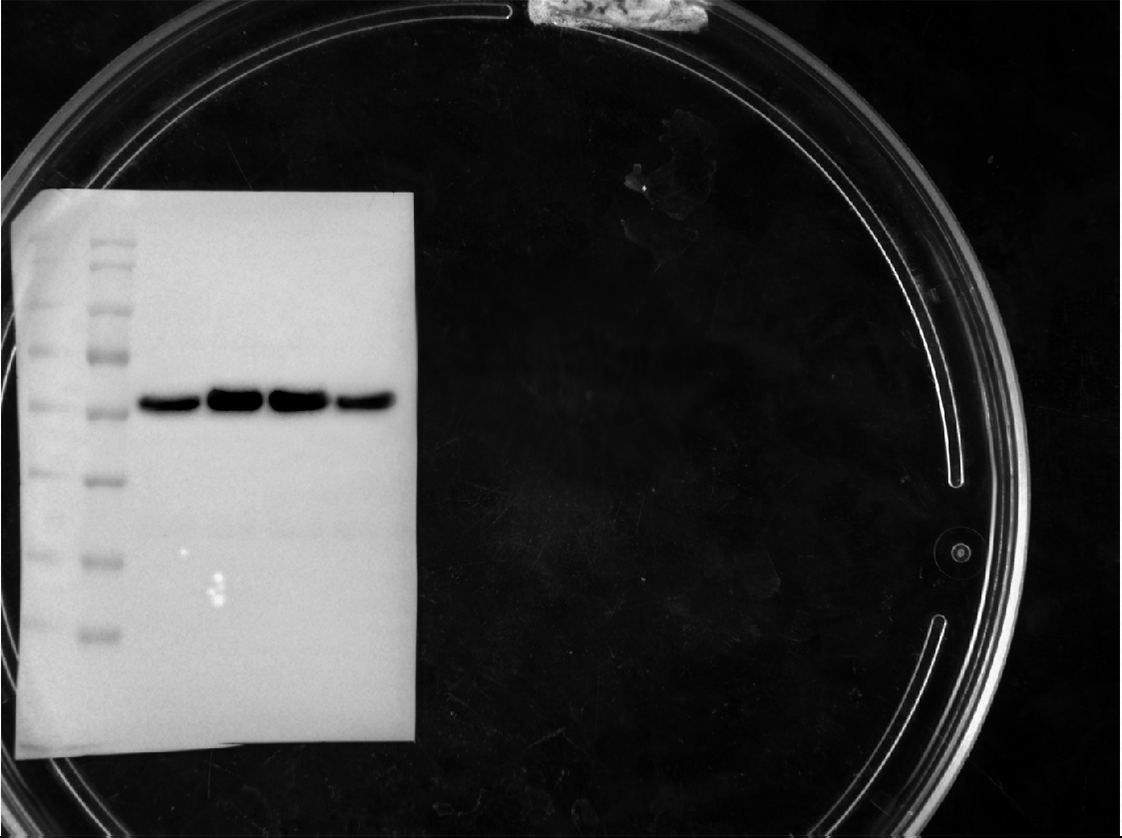

Supplement: Supplemental Information 2 [file peerj-11-15840-s002.zip › Western blot/figure 5 Mammalian STE20-like kinase 1 (MST1)/Original.tif]

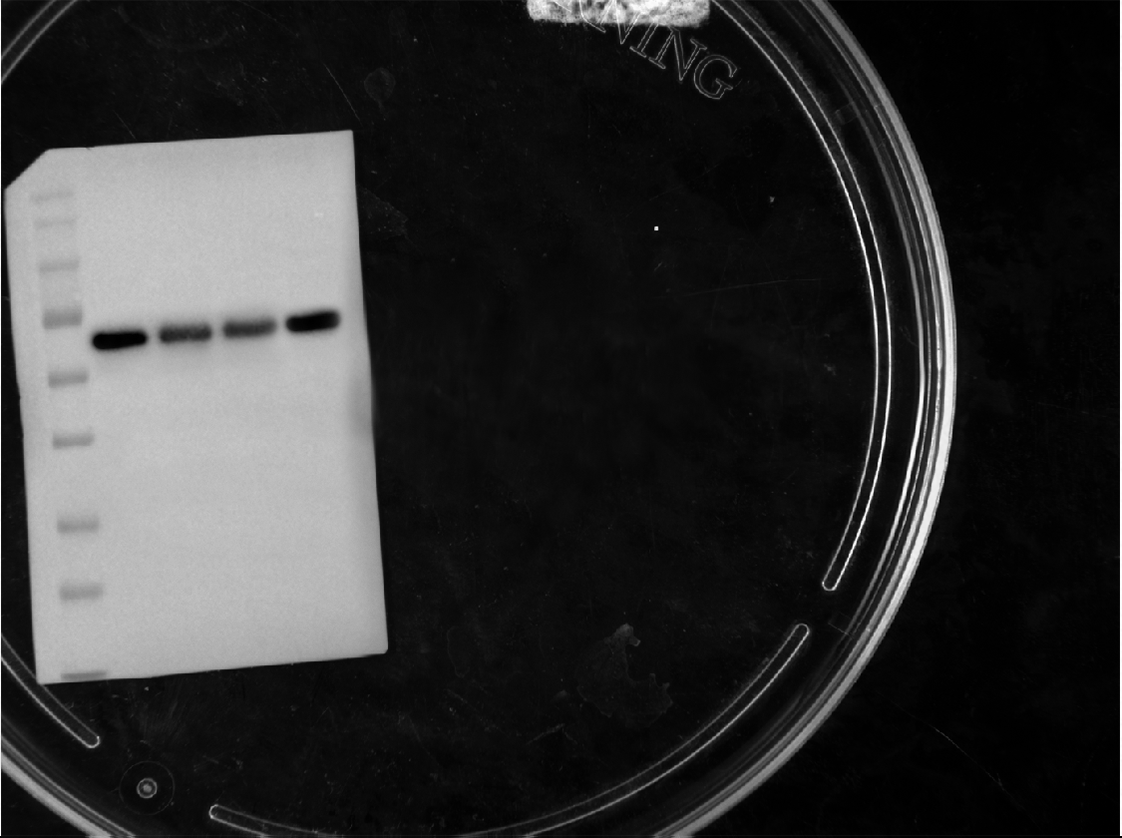

Supplement: Supplemental Information 2 [file peerj-11-15840-s002.zip › Western blot/figure 5 P-Adenosine 5-monophosphate (AMP)-activated protein kinase(P-AMPK)/Original.tif]

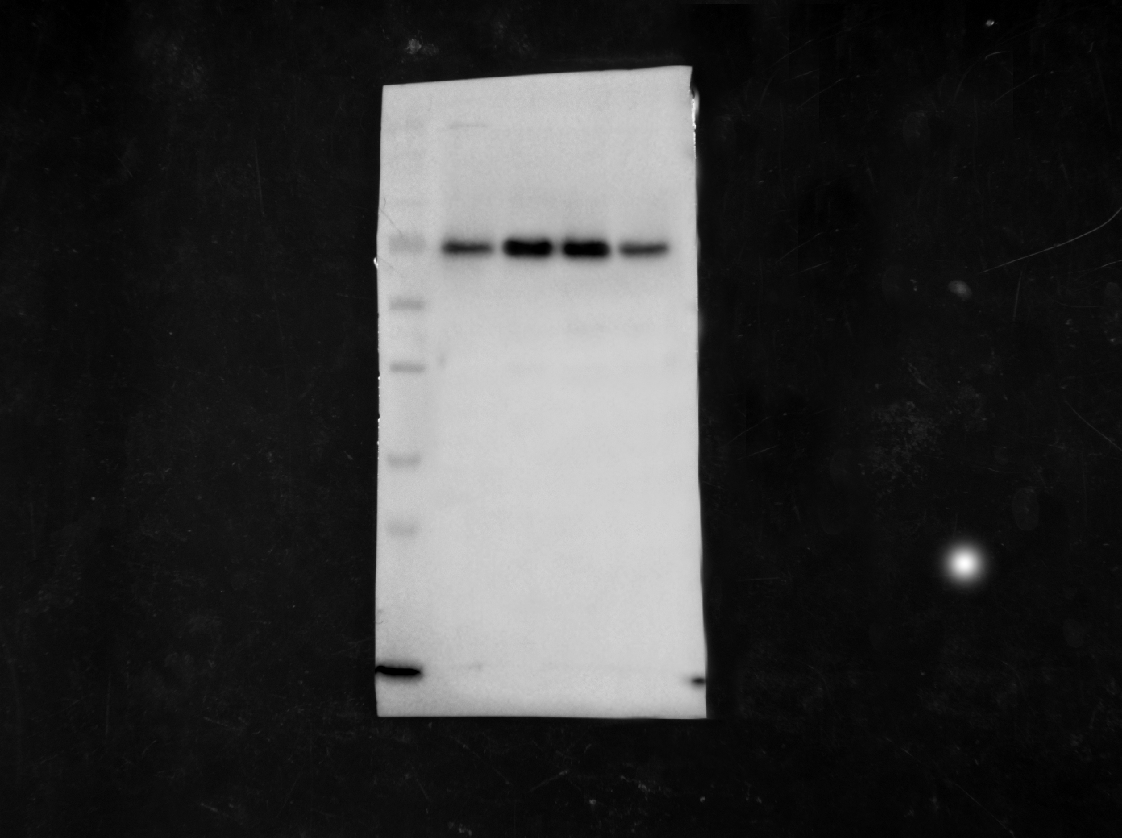

Supplement: Supplemental Information 2 [file peerj-11-15840-s002.zip › Western blot/figure 5 p-Forkhead box O3 (p-FOXO3)/Original.tif]

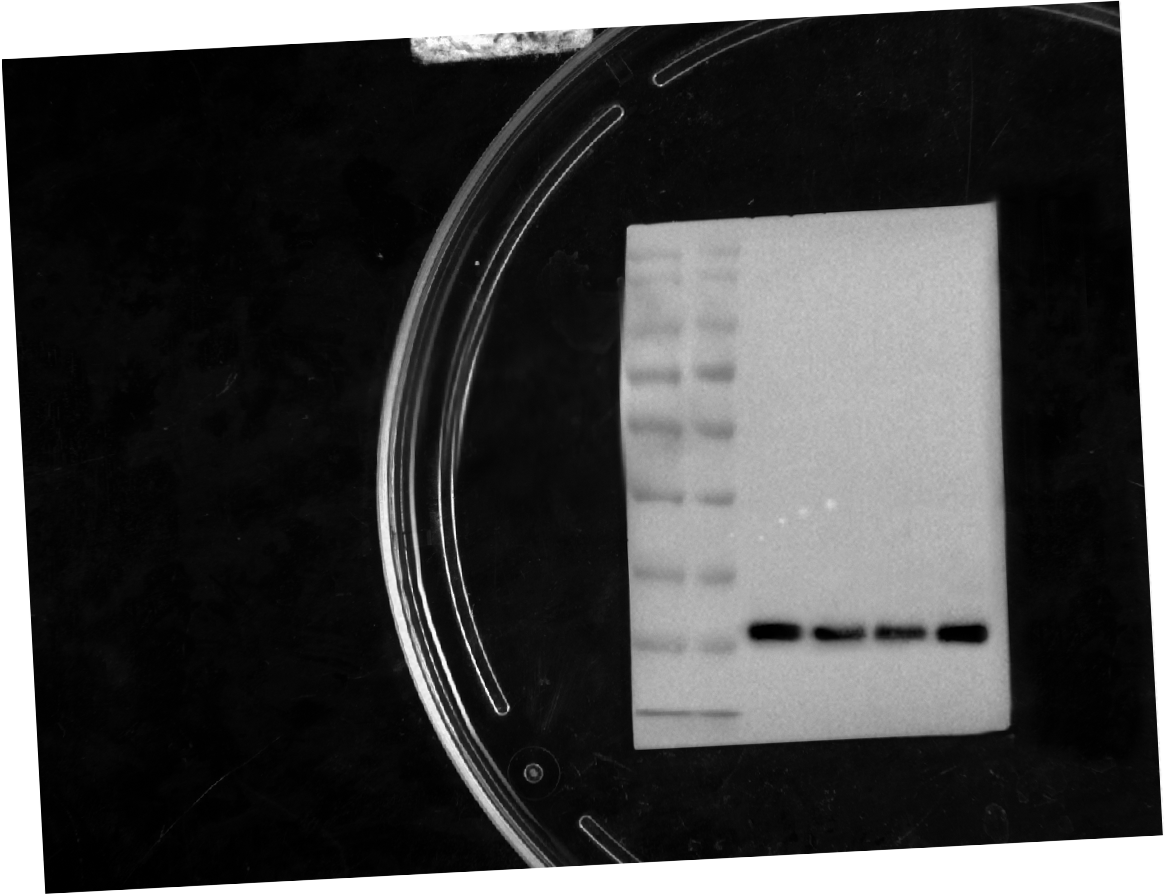

Supplement: Supplemental Information 2 [file peerj-11-15840-s002.zip › Western blot/figure 5 Sirtuin 3 (SIRT3)/Original.tif]

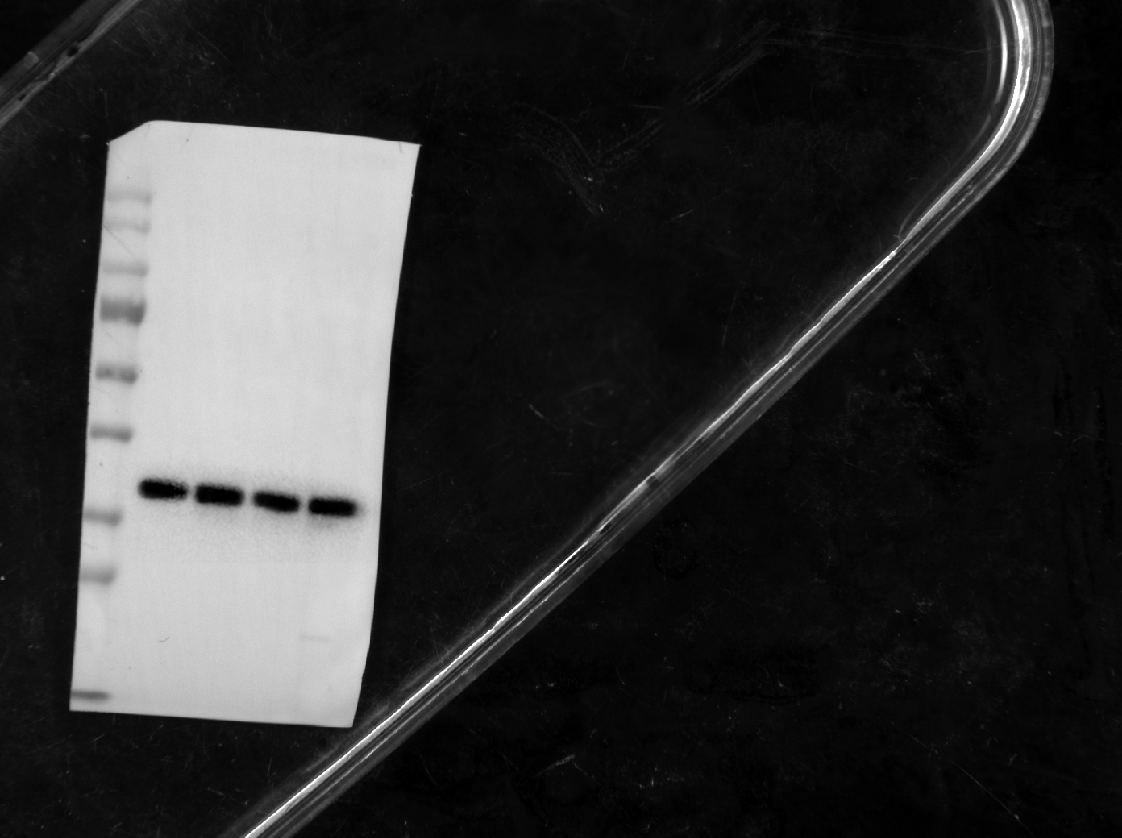

Supplement: Supplemental Information 2 [file peerj-11-15840-s002.zip › Western blot/glyceraldehyde-3-phosphate dehydrogenase (GAPDH)/Original.tif]
