# Supplementary material for: miR-200a-3p overexpression alleviates diabetic cardiomyopathy injury in mice by regulating autophagy through the FOXO3/Mst1/Sirt3/AMPK axis
Source: PeerJ. 2023 Sep 15;11:e15840. doi: 10.7717/peerj.15840 (PMC10506579; doi:10.7717/peerj.15840)

**Control group**


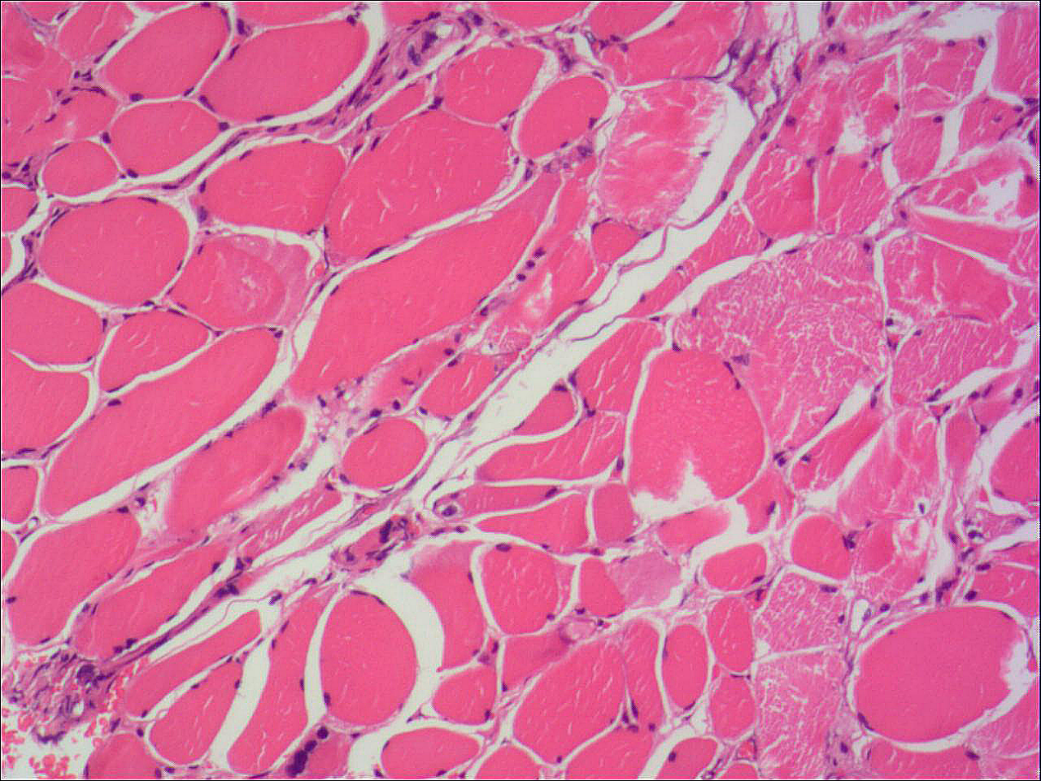


**DCM group**


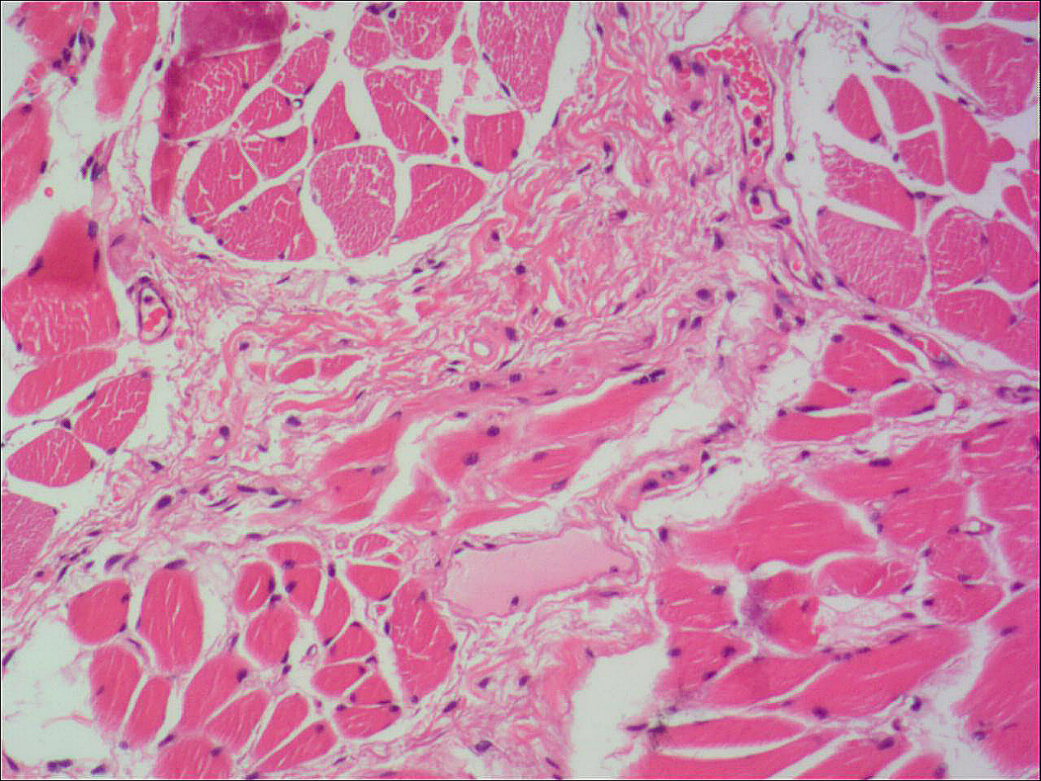


**DCM+rAAV-SC**


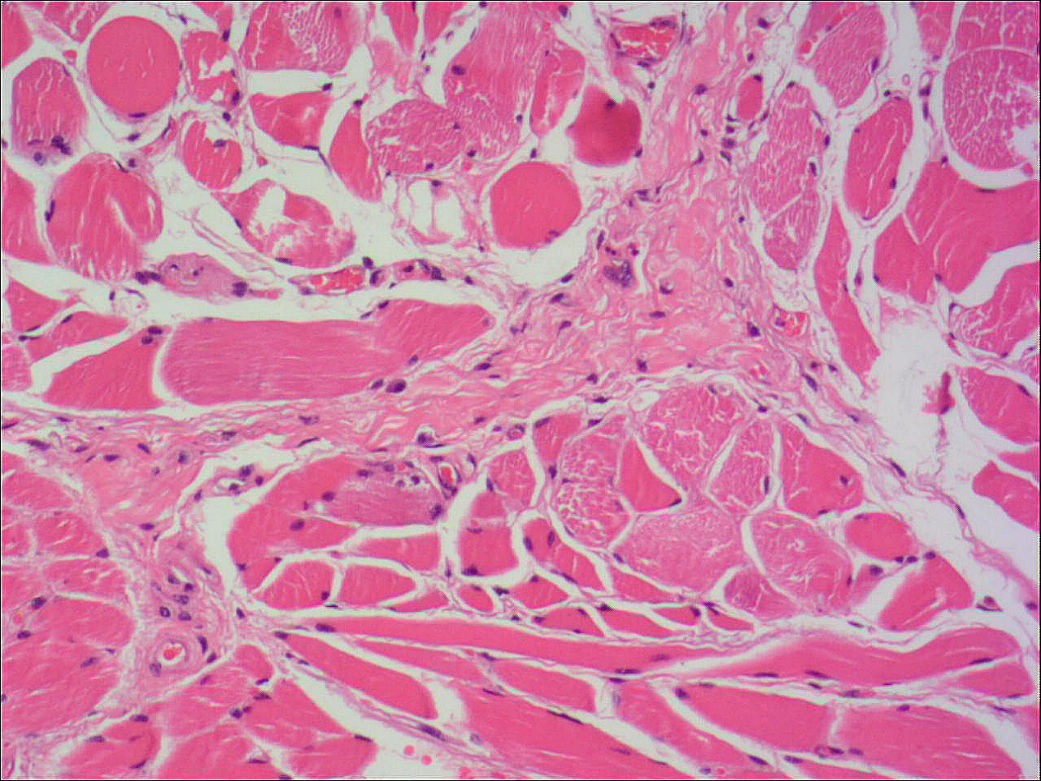


**DCM+rAAV-miR-200a-3p**


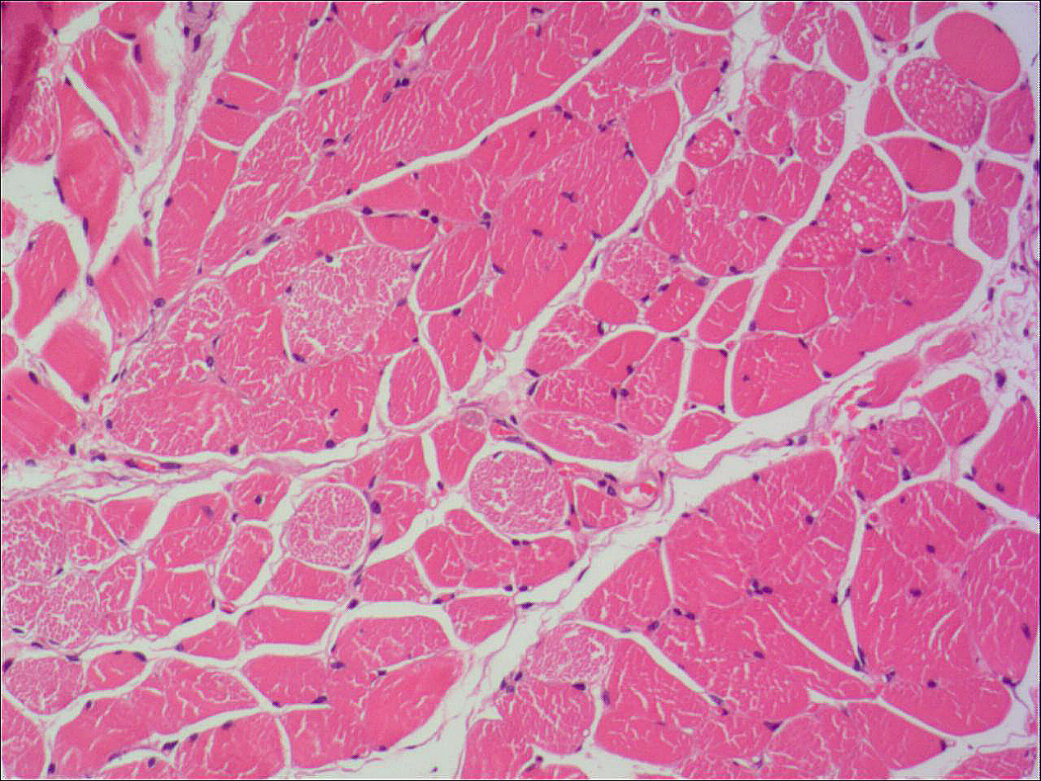


**Control group**


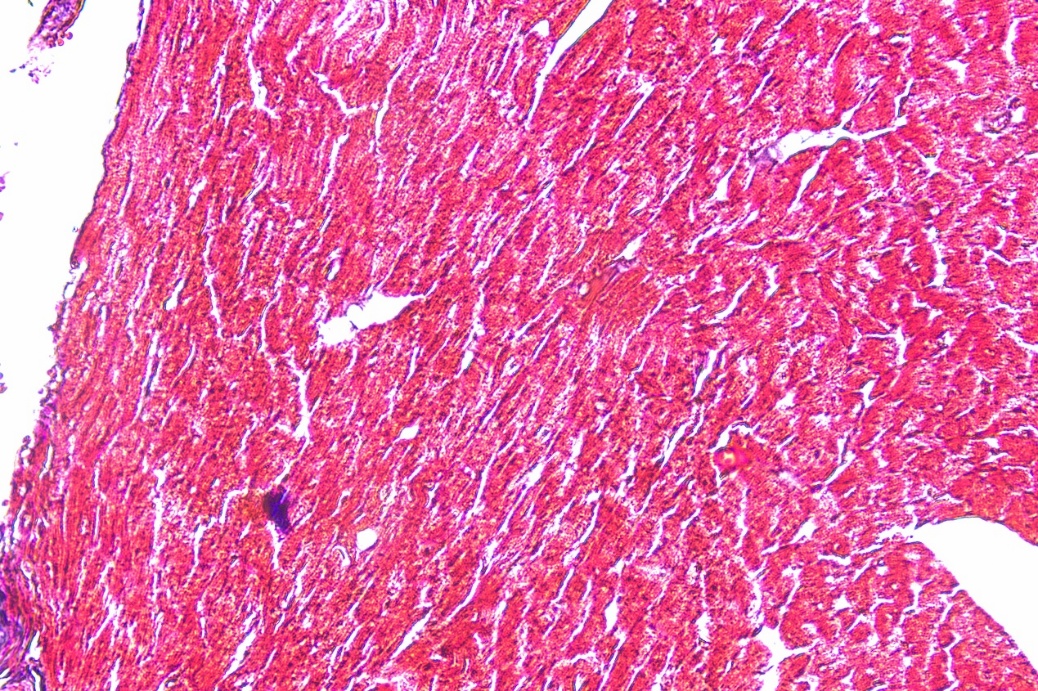


**DCM group**


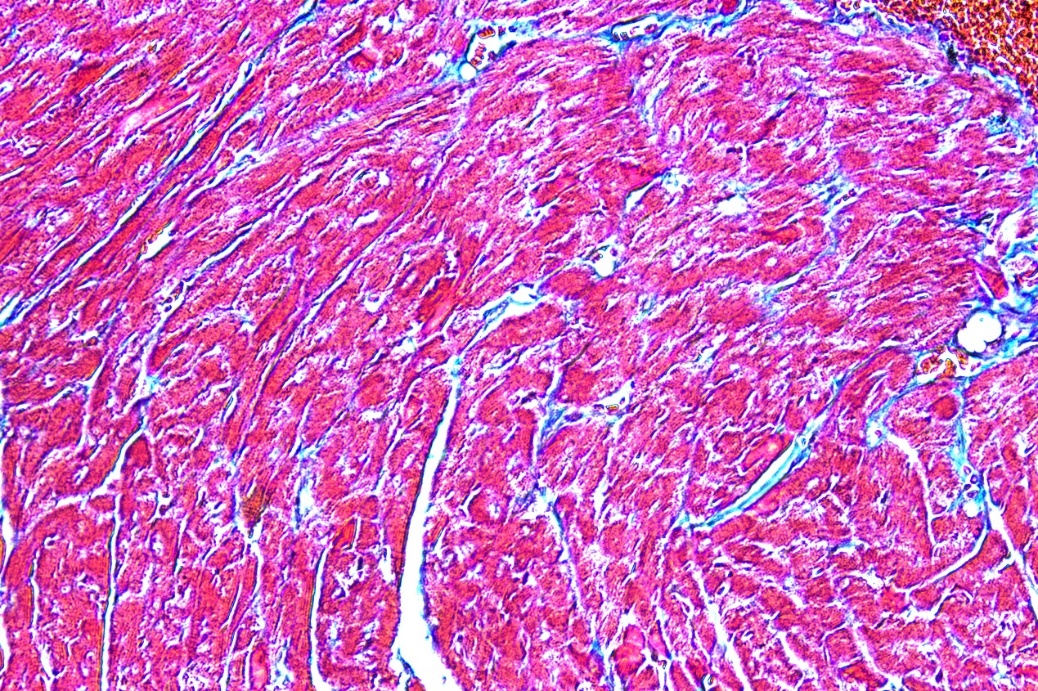


**DCM+rAAV-SC**


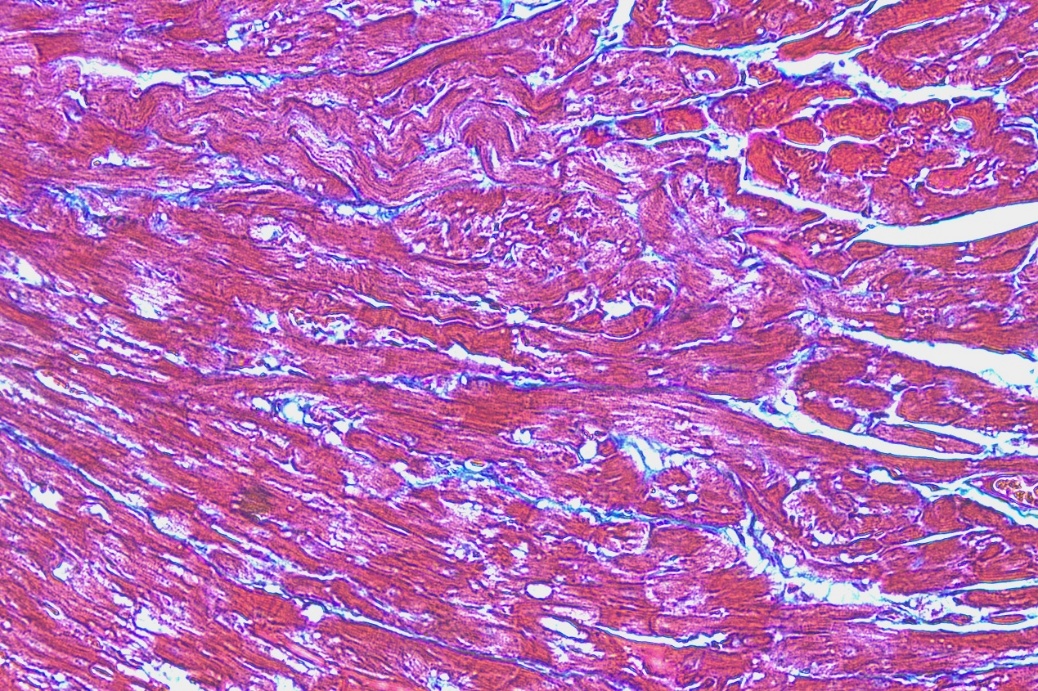


**DCM+rAAV-miR-200a-3p**


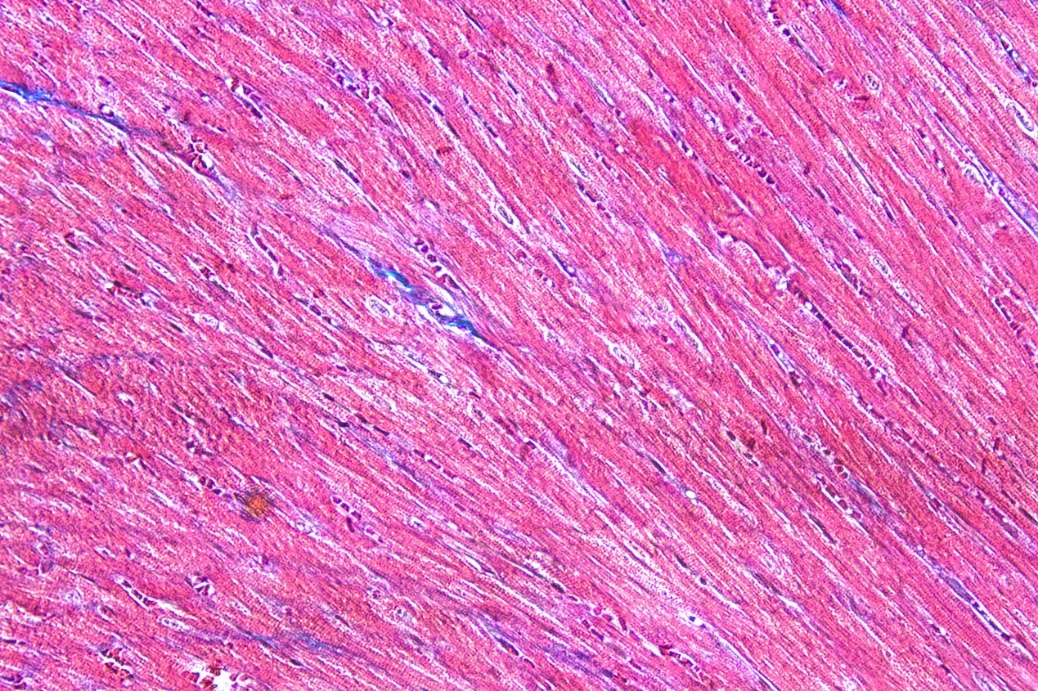


**Control group**


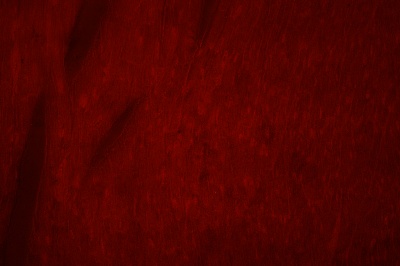

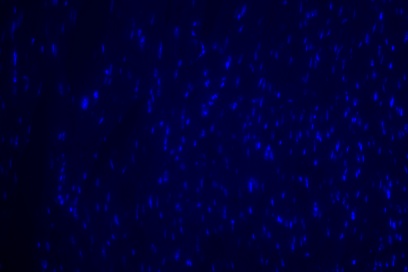

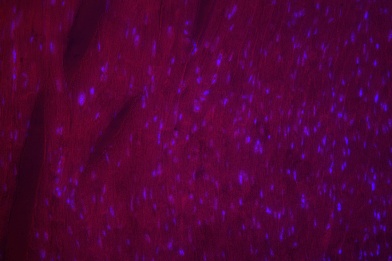


**DCM group**


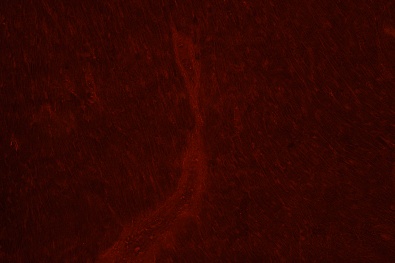

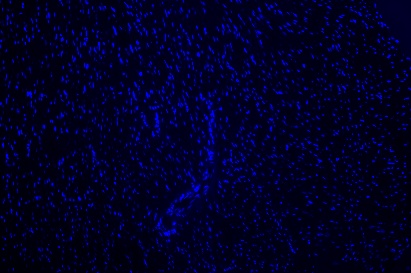

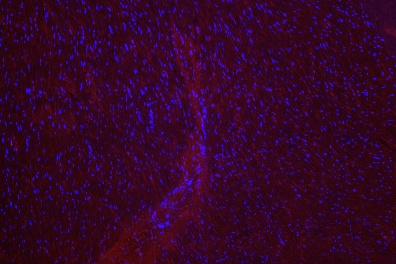


**DCM+rAAV-SC**


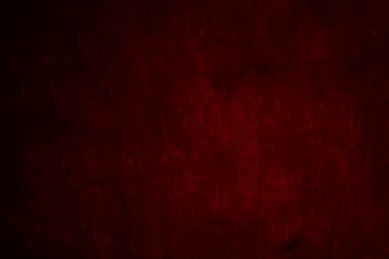

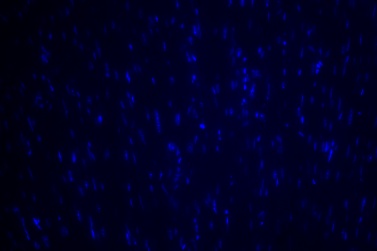

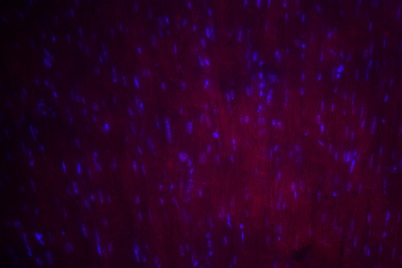


**DCM+rAAV-miR-200a-3p**


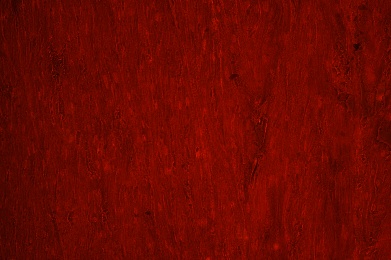

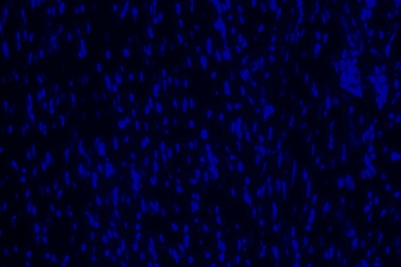

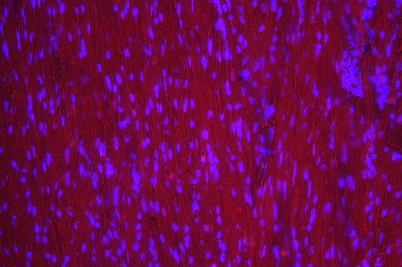


**Control group**


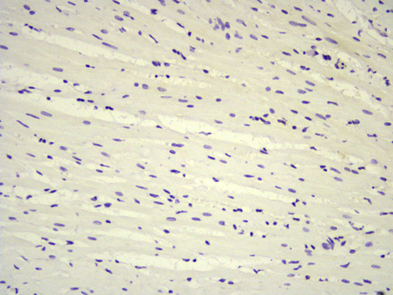


**DCM group**


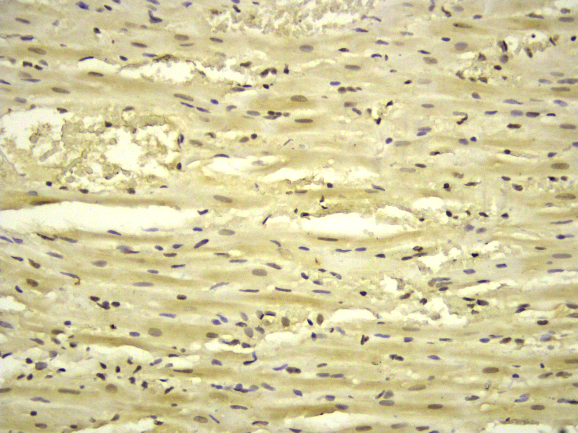


**DCM+rAAV-SC**


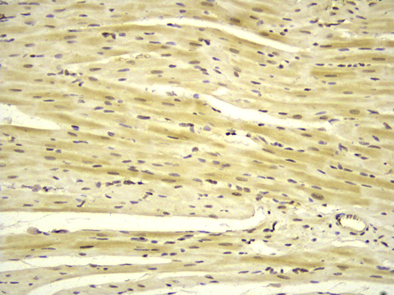


**DCM+rAAV-miR-200a-3p**


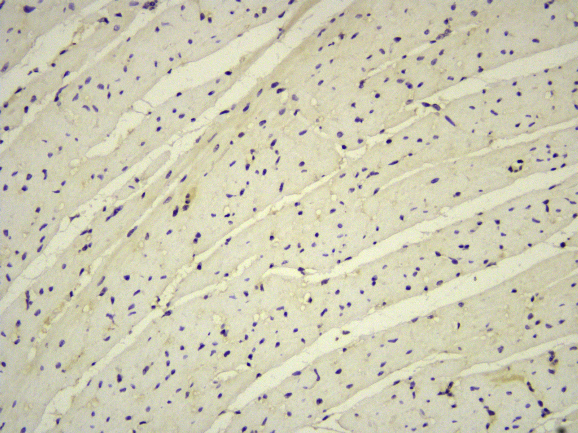


LC3 II/I


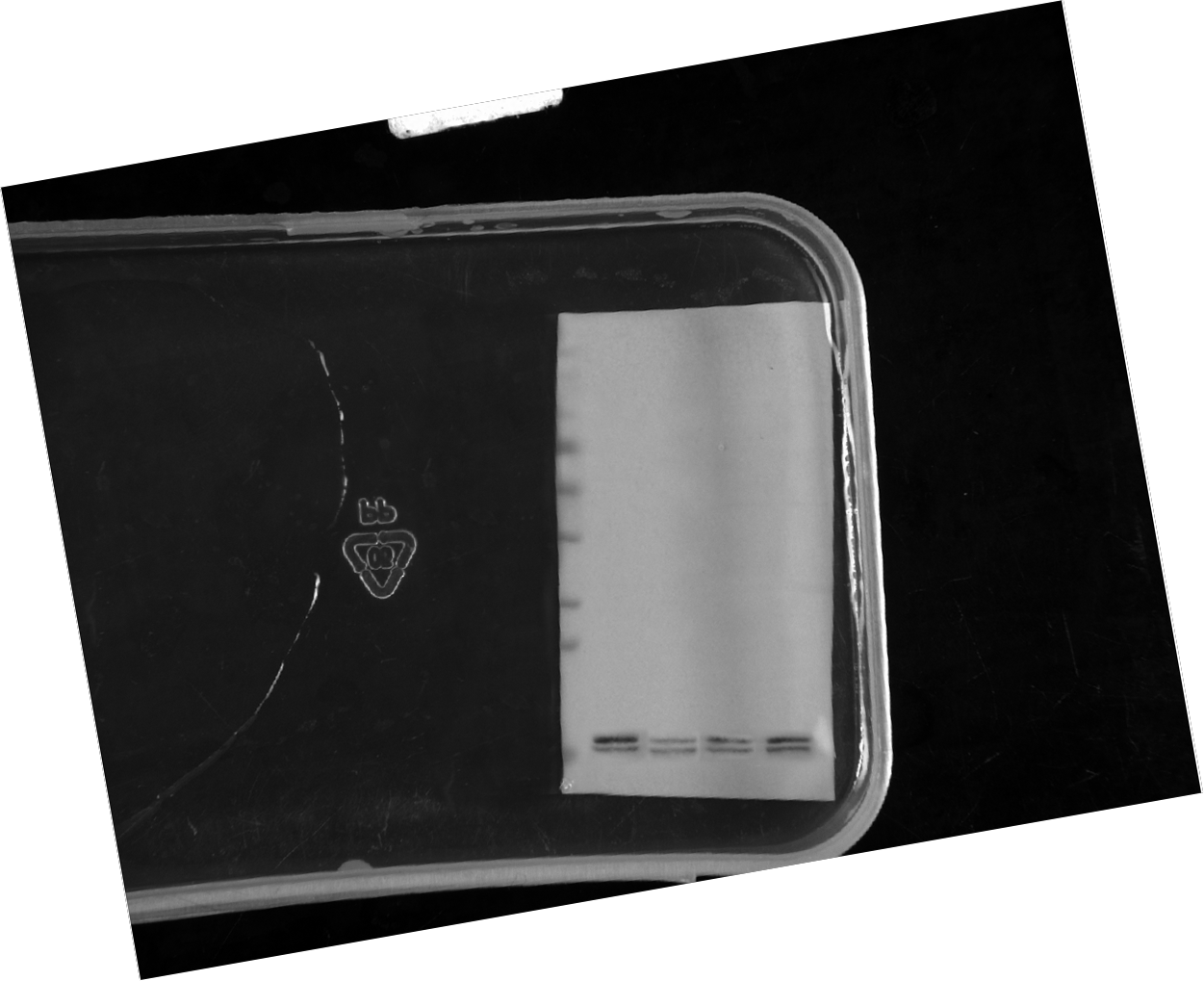


Beclin 1


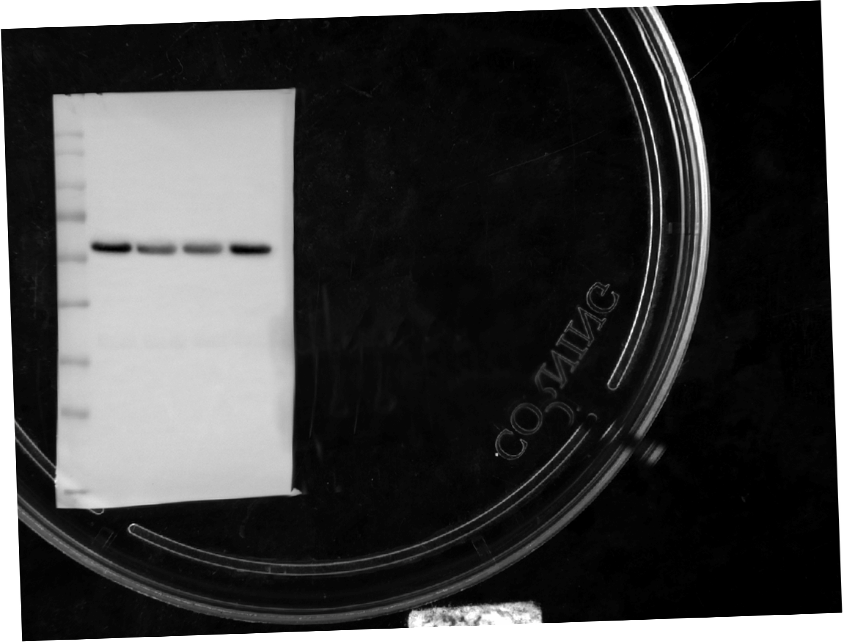


Caspase-3


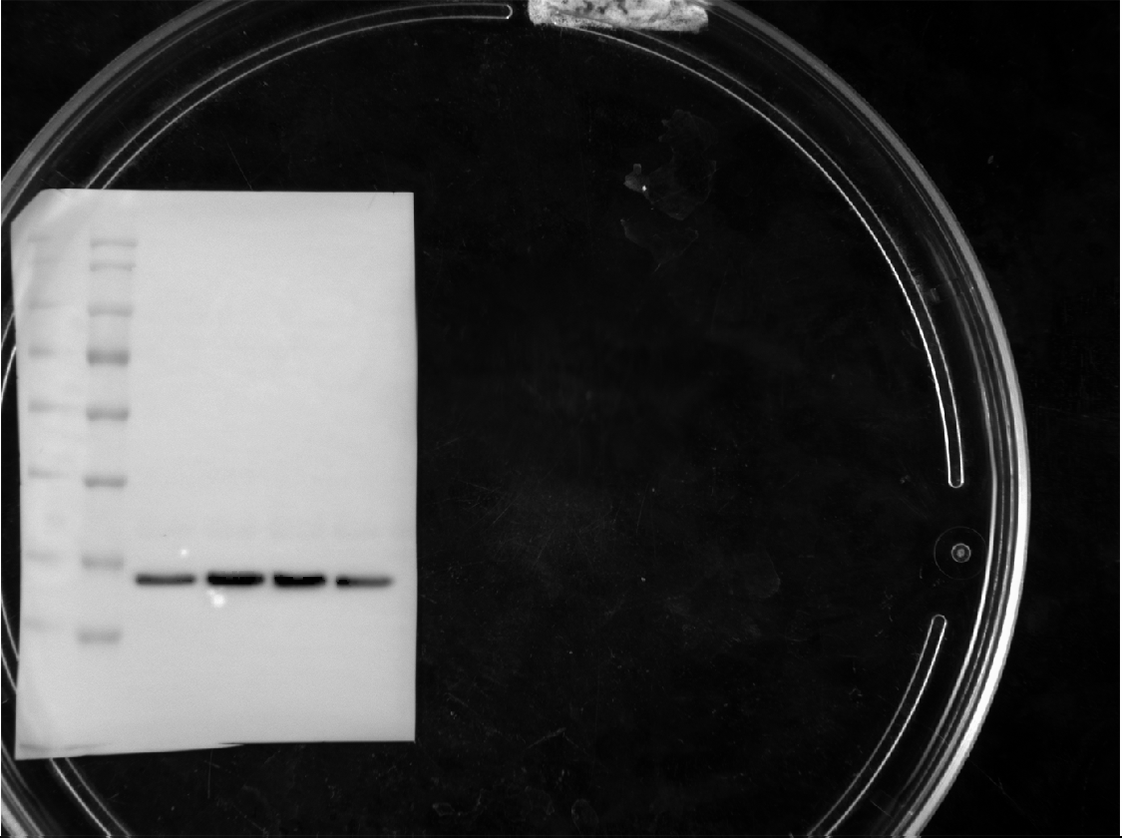


p-FOXO3


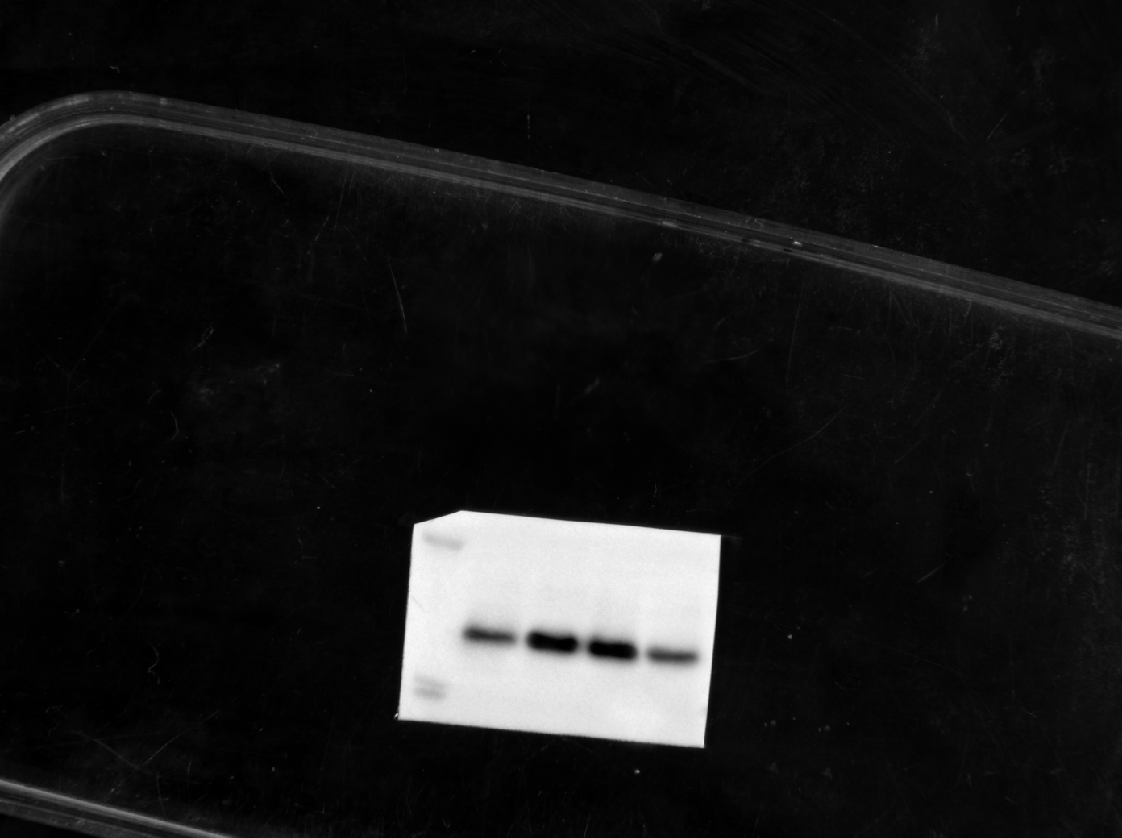


FOXO3


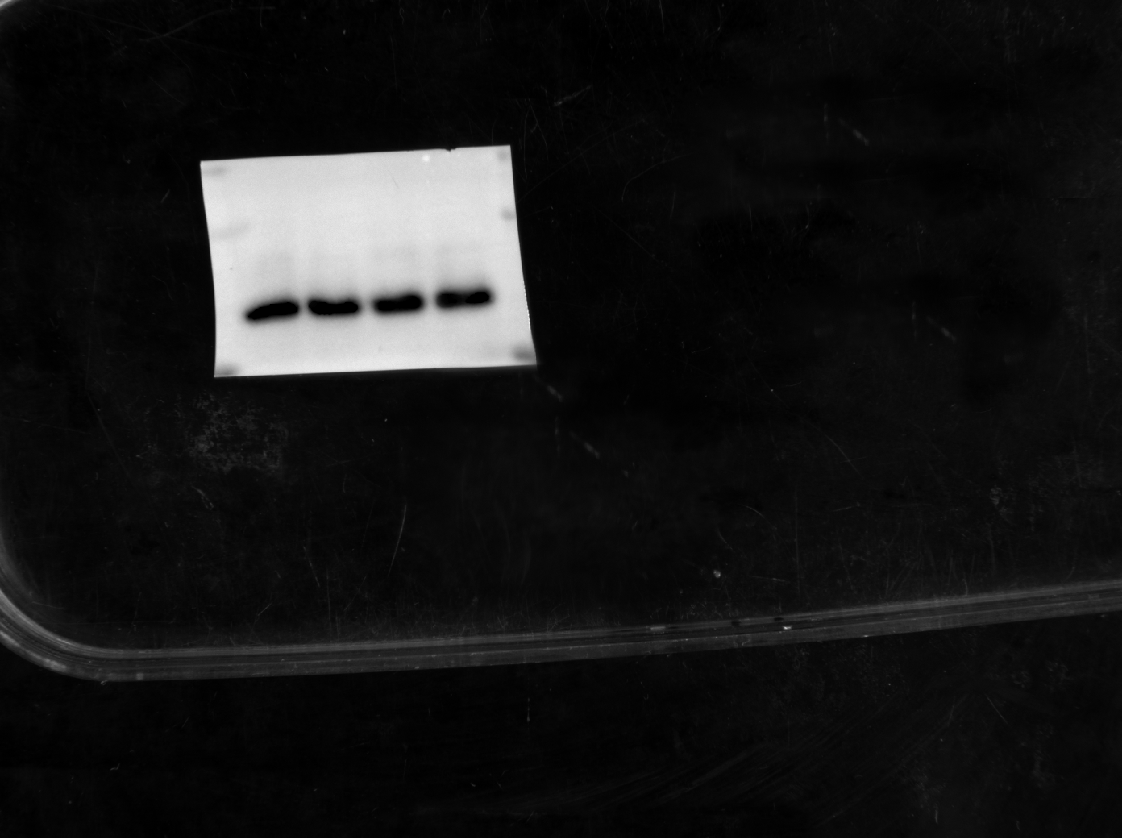


MST1


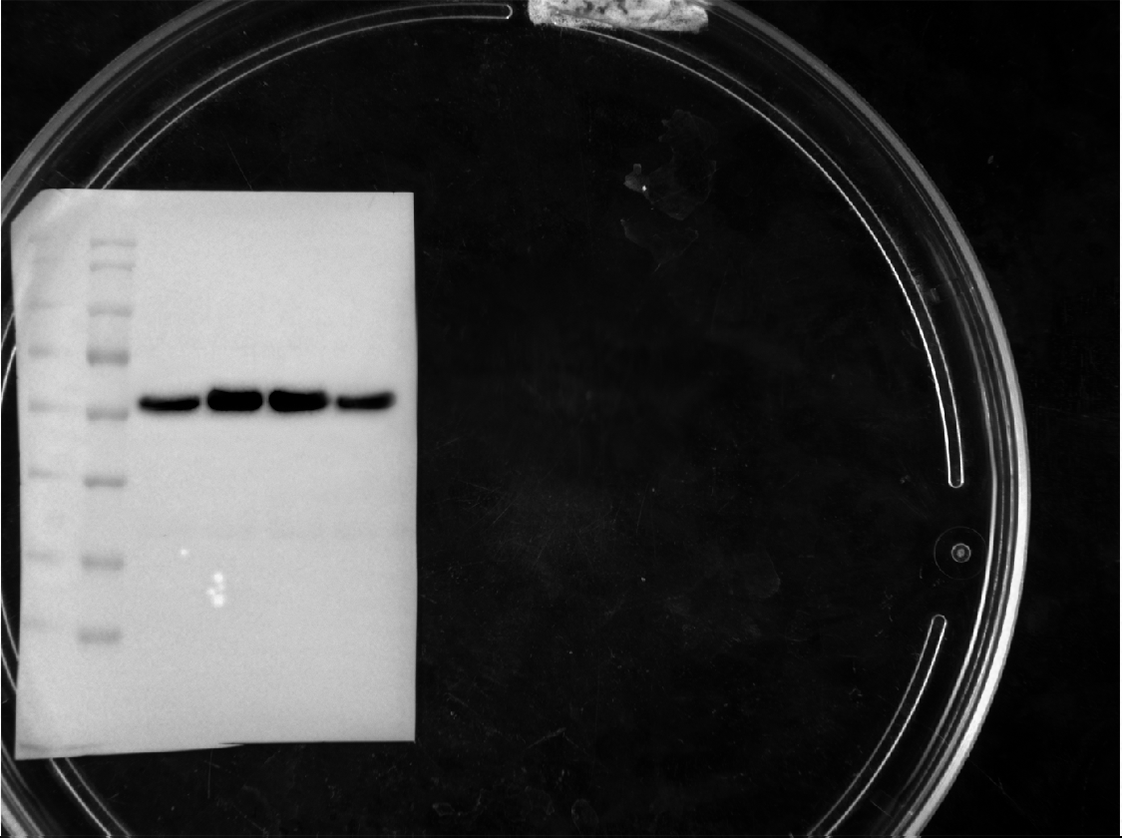


Sirt3


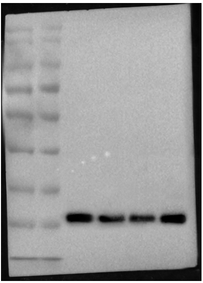


p-AMPK


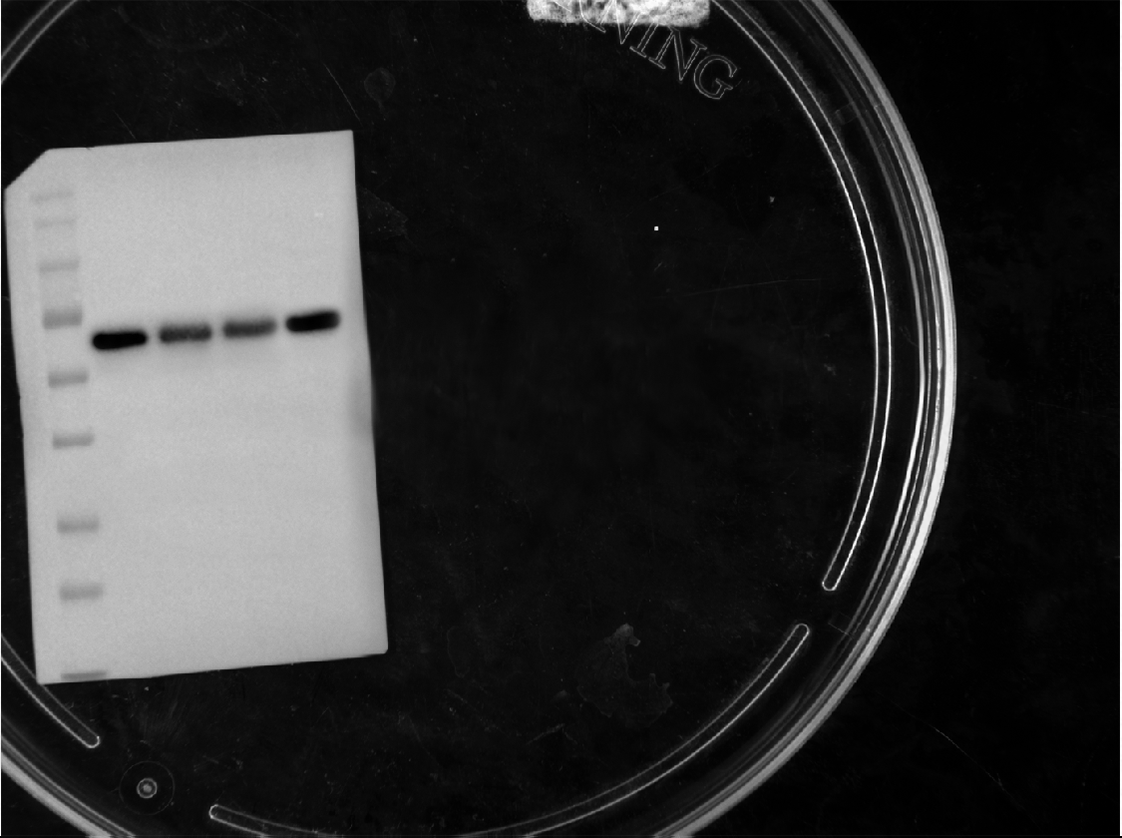


AMPK


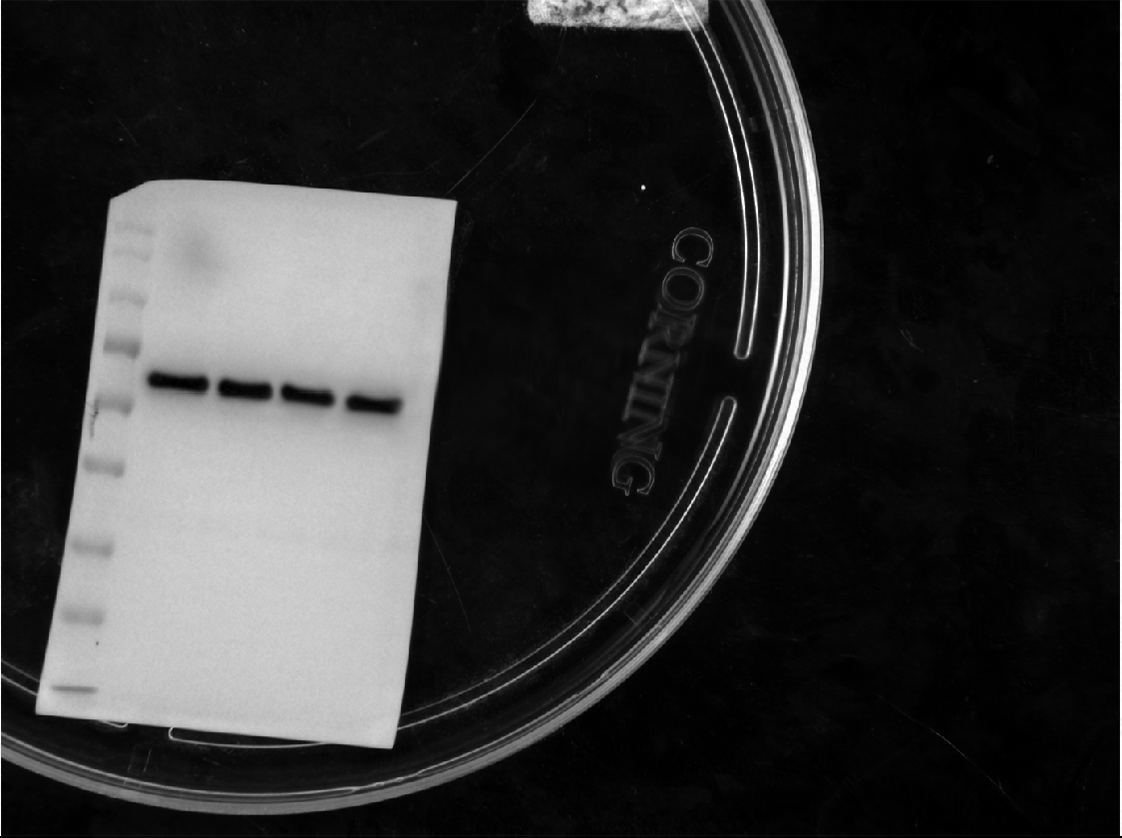


GAPDH


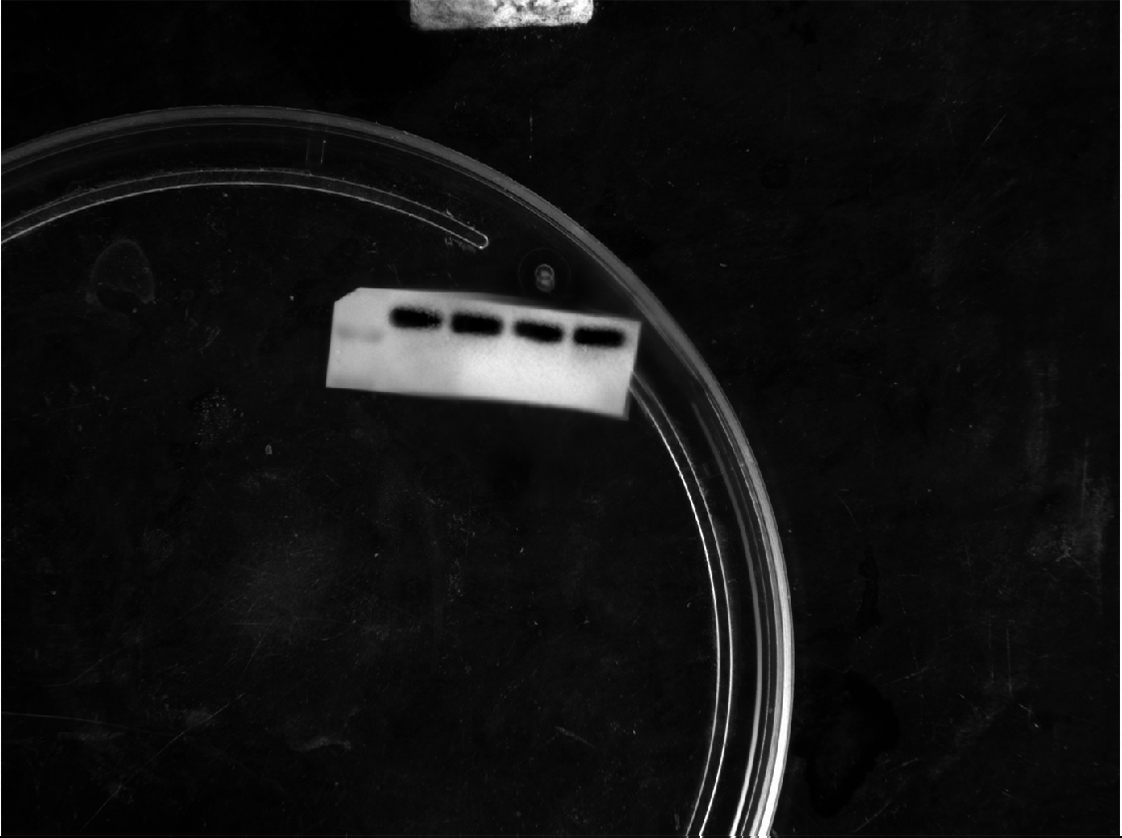

Supplement: Supplemental Information 4 [file peerj-11-15840-s004.docx]
